# Incidence and Outcomes of Patients With Early Cardiac Complications After Intracerebral Hemorrhage: A Report From VISTA

**DOI:** 10.1161/STROKEAHA.124.048189

**Published:** 2024-10-02

**Authors:** Hironori Ishiguchi, Bi Huang, Wahbi K. El-Bouri, Jesse Dawson, Gregory Y.H. Lip, Azmil H. Abdul-Rahim

**Affiliations:** 1 Liverpool Centre for Cardiovascular Science at University of Liverpool, Liverpool John Moores University and Liverpool Heart and Chest Hospital, United Kingdom (H.I., B.H., W.K.E.-B., G.Y.H.L., A.H.A.-R.).; 2 Division of Cardiology, Department of Medicine and Clinical Science, Yamaguchi University Graduate School of Medicine, Ube, Japan (H.I.).; 3 Department of Cardiovascular and Metabolic Medicine, Institute of Life Course and Medical Sciences, University of Liverpool, United Kingdom (H.I., B.H., W.K.E.-B., A.H.A.-R.).; 4 School of Cardiovascular and Metabolic Health, College of Medical, Veterinary and Life Sciences, University of Glasgow, United Kingdom (J.D.).; 5 Department of Clinical Medicine, Danish Centre for Health Services Research, Aalborg University, Denmark (G.Y.H.L.).; 6 Stroke Division, Department Medicine for Older People, Mersey and West Lancashire Teaching Hospitals NHS Trust, Prescot, United Kingdom (A.H.A.-R.).

**Keywords:** atrial fibrillation, cerebral hemorrhage, heart failure, intracranial hemorrhages, ischemic stroke

## Abstract

**BACKGROUND:**

The incidence and outcomes of early cardiac complications in patients with intracerebral hemorrhage (ICH) are poorly understood. These cardiac complications may be part of the so-called stroke-heart syndrome in patients with ICH. We investigated this issue in an individual patient data pooled analysis from an international repository of clinical trial data.

**METHODS:**

We used the Virtual International Stroke Trials Archive to investigate the incidence of cardiac complications within 30 days post-ICH or acute ischemic stroke (AIS). These complications included acute coronary syndrome encompassing myocardial injury, heart failure/left ventricular dysfunction, atrial fibrillation/atrial flutter, other arrhythmia/ECG abnormalities, and cardiorespiratory arrest. We used propensity score matching to compare the incidence of patients with stroke-heart syndrome in patients with ICH with those following AIS. Factors associated with 90-day mortality were evaluated using multivariate logistic regression analysis in the ICH cohort.

**RESULTS:**

We pooled data from 8698 participants recruited in acute stroke trials (mean age, 68±12 years; 56% male), of whom 914 (11%) were patients with ICH. Among the patients with ICH, 123 (13%) had stroke-heart syndrome in patients with ICH. Following propensity score matching, a total of 1828 patients (914 for each of the cohorts) were analyzed. While the overall incidence of cardiac events tended to be lower in the ICH group compared with the AIS group (the cumulative incidence freedom from the event, 86.3% [95% CI, 84.1–88.6] versus 83.6% [95% CI, 81.2–86.0]; *P*=0.100), the incidences cardiac events other than atrial fibrillation/atrial flutter were comparable between the 2 matched groups. The incidence of atrial fibrillation/atrial flutter was significantly lower in the ICH group than in the AIS group (*P*<0.001). The multivariate-adjusted analysis found that stroke-heart syndrome in patients with ICH was associated with 90-day mortality (adjusted odds ratio, 1.12 [95% CI, 1.06–1.19]; *P*<0.001).

**CONCLUSIONS:**

Cardiac events are common and negatively affect prognosis in patients with ICH, just as seen in AIS.

Intracerebral hemorrhage (ICH) is the second most common subtype of stroke, following acute ischemic stroke (AIS).^1,2^ ICH still has a poor clinical outcome, and many challenges remain.^2,3^ Notably, the incidence of complications during hospitalization in patients with ICH has been increasing, with ≈30% experiencing some form of complication.^4^

Cardiac complications occurring during the acute phase of an ischemic stroke have been collectively termed stroke-heart syndrome (SHS).^5,6^ SHS represents the development of new cardiac abnormalities or the exacerbation of preexisting heart diseases within 30 days following the onset of AIS. Clinical manifestations of SHS include acute myocardial infarction (MI), acute myocardial injury, heart failure (HF), and left ventricular (LV) dysfunction such as Takotsubo syndrome, arrhythmias (including ECG abnormalities and atrial fibrillation [AF]), and sudden cardiac death. The pathophysiology of SHS is largely attributed to autonomic dysregulation, neurohormonal disturbances, and neuroinflammatory reactions, which arise from the impaired interactions between the heart and brain due to cerebral damage.^7–9^

Cardiac complications represent ≈20% of all complications and are the second leading cause of mortality in patients with AIS.^10^ Moreover, patients with SHS experience both short- and long-term worse prognoses. However, the incidence and prognostic impact of these events on patients with ICH still remain uncertain.^11,12^ In this study, we aimed to assess the incidence and prognostic impact of cardiac events in patients with ICH.

## METHODS

### Data Resource and Ethical Information

We conducted a retrospective analysis of the cohort data from the Virtual International Stroke Trials Archive (VISTA) resource (https://www.virtualtrialsarchives.org/vista/).^13^ VISTA serves as a collaborative platform, collecting patient data from completed acute stroke trials (from the year 1998 to 2010), anonymized in relation to patients and trials’ identity, for novel exploratory analyses.^14^ VISTA has institutional ethical approval (University of Glasgow United Kingdom, College of Medical Veterinary and Life Sciences’ Ethics Committee) for the use of fully anonymized data for novel research purposes. Informed consent was not sought for the present study because it uses pooled, anonymized data from a clinical trials resource. Anonymized patient-level data from VISTA are available upon reasonable request through its online platform.

All patients with stroke were treated as per institutional practice and stroke guidelines acceptable at the point of trial conduct.^14^ The conduct and reporting of our analysis are in accordance with the Strengthening the Reporting of Observational Studies in Epidemiology guidelines for cohort studies.^15^ We selected patients with ICH and AIS who had been randomized to receive a placebo or a drug now known to have no confirmed effect on stroke outcomes. We included patients for whom we had baseline demographics and outcome information.

### Study Design

Our study was designed to compare the incidence of cardiac events during the acute phase between patients with ICH and those with AIS. Given the significant differences in initial patient profiles, we applied propensity score matching (PSM) to align the groups more closely. Any patients with incomplete demographic data, which were essential for the PSM, were excluded from the analysis. Following PSM, we utilized Kaplan-Meier analysis to assess the cumulative incidences. Furthermore, to evaluate the impact of cardiac events on 90-day mortality in patients with ICH, we conducted a multivariate analysis of the cohort.

### Stroke-Heart Syndrome

SHS was defined in patients with AIS as the development of at least one of the cardiac manifestations within 30 days of stroke onset: acute coronary syndrome (ACS; including acute MI and unstable angina pectoris)/myocardial injury (indicated by terms such as myocardial enzyme elevation or increase in troponin), HF/LV dysfunction, AF/atrial flutter (AFL), other arrhythmia/ECG abnormalities (primarily QTc prolongation), and cardiorespiratory arrest. In this study, we also classified patients with ICH who developed any cardiac events within 30 days of stroke onset as SHS in patients with ICH (SHS-ICH).

The diagnosis was established by identifying definitive cardiac events in the list of adverse events in the database. To ensure accuracy, data collection was independently undertaken by 2 authors (H.I. and B.H.), who cross-verified their findings for consistency.

### Propensity Score Matching

PSM was executed using the nearest neighbor matching technique, aligning the 2 groups on a 1:1 ratio based on age, sex, initial National Institutes of Health Stroke Scale scores, systolic and diastolic blood pressures, hypertension, diabetes, a history of stroke, AF and MI, estimated glomerular filtration rate level, and hemoglobin values. Although the original database included information on the history of HF, this variable was not used in the PSM due to a high number of missing values (>50%). We performed a sensitivity analysis using a PSM model that included the history of HF to compare the incidence of cardiac events. Standardized mean differences were evaluated to assess the balance between groups before and after matching. We compared the standardized mean difference in each variable between PSM and inverse probability weighting with standardized weights calculated from the propensity score and adopted the method that provided better balance.

### Statistical Analysis

Variables conforming to normal distributions were depicted as mean±SD, while those deviating from normality were presented as medians with interquartile ranges. Differences in modified Rankin Scale scores were calculated using the *t* test. The cumulative incidence of freedom from SHS and each manifestation, along with 95% CI, was calculated. The log-rank test was performed to assess differences between cohorts. The χ^2^ tests were used to compare the differences in the proportions of arrhythmia subcategories between groups.

In the ICH cohort, a multivariate analysis was conducted using logistic regression, with the 90-day mortality designated as the dependent variable. The model incorporated covariates such as age, sex, initial National Institutes of Health Stroke Scale score, systolic blood pressure, comorbidities (including hypertension, diabetes, history of stroke, MI, and AF), and laboratory findings (hemoglobin and estimated glomerular filtration rate levels). Results were expressed as an odds ratio with 95% CI. For the sensitivity analysis, we also performed the multivariate analysis on the entire ICH cohort, incorporating ICH volume as an additional covariate. A *P* value of <0.05 was considered statistically significant. All analyses were performed using R, version 4.3.0, on a virtual network computer provided by VISTA.

## RESULTS

### Patient Demographics

**Figure 1.**
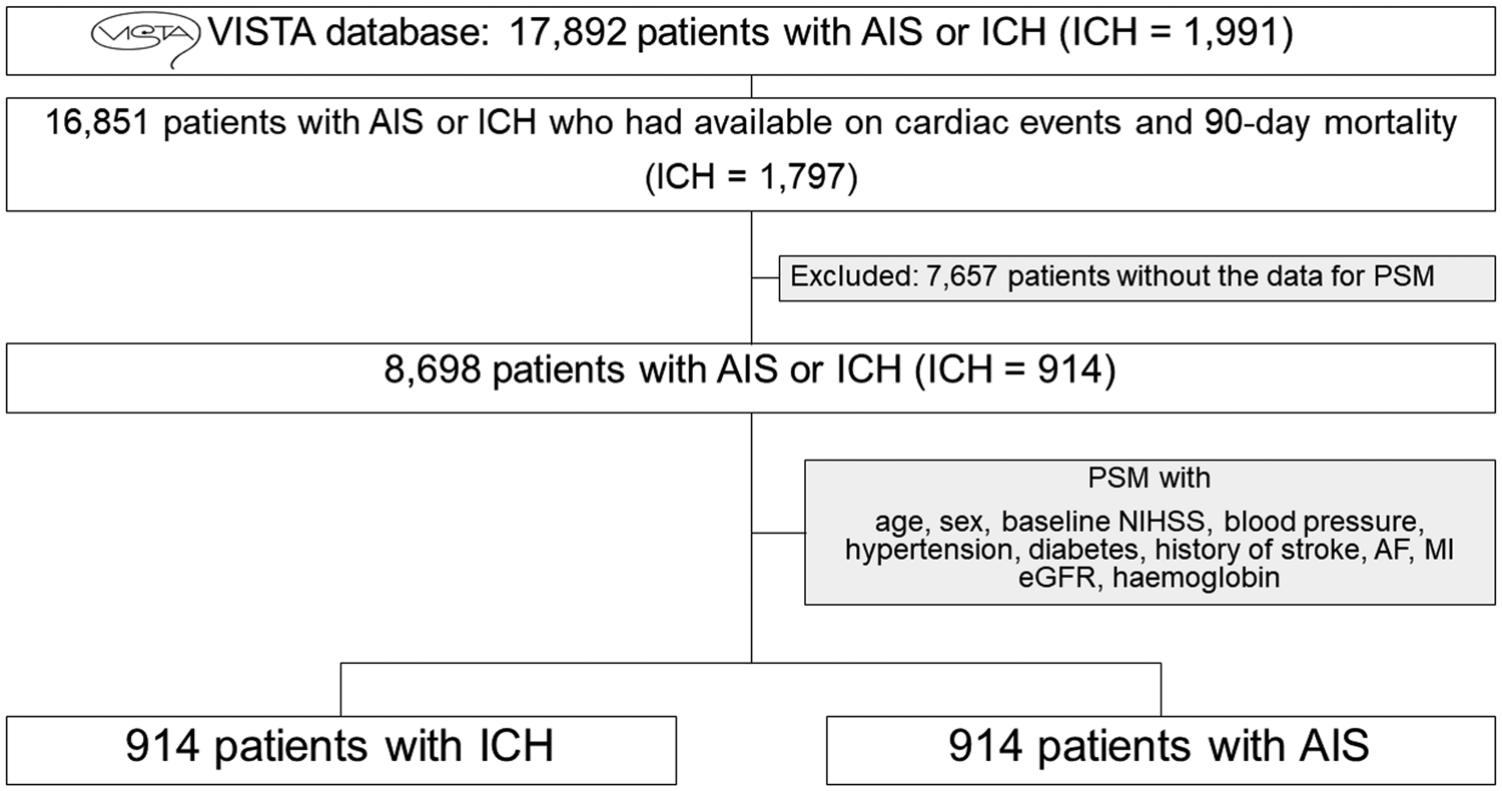
**Study flow diagram.** AF indicates atrial fibrillation; AIS, acute ischemic stroke; eGFR, estimated glomerular filtration rate; ICH, intracranial hemorrhage; MI, myocardial infarction; NIHSS, National Institutes of Health Stroke Scale; PSM, propensity score matching; and VISTA, Virtual International Stroke Trials Archive.

Figure 1 presents the flowchart of this study. From an initial pool of 17 892 patients, 16 851 had available outcome information. Patient demographics for the whole cohort are shown in Table S1. Patients with ICH had higher systolic and diastolic blood pressures and a lower prevalence of cardiac comorbidities (AF, MI, and HF). Of those, 8698 with complete data for PSM were identified. Following PSM, 914 patients from each group were selected. The standardized mean difference for each variable was mostly lower with PSM than with inverse probability weighting (Figure S1). Therefore, we chose to conduct the analysis using PSM. All patients with ICH before PSM were retained in the cohort after PSM. Demographic details of the patients after PSM are shown in Table 1. The standardized mean differences for all variables were maintained at <0.1.

**Table 1.**
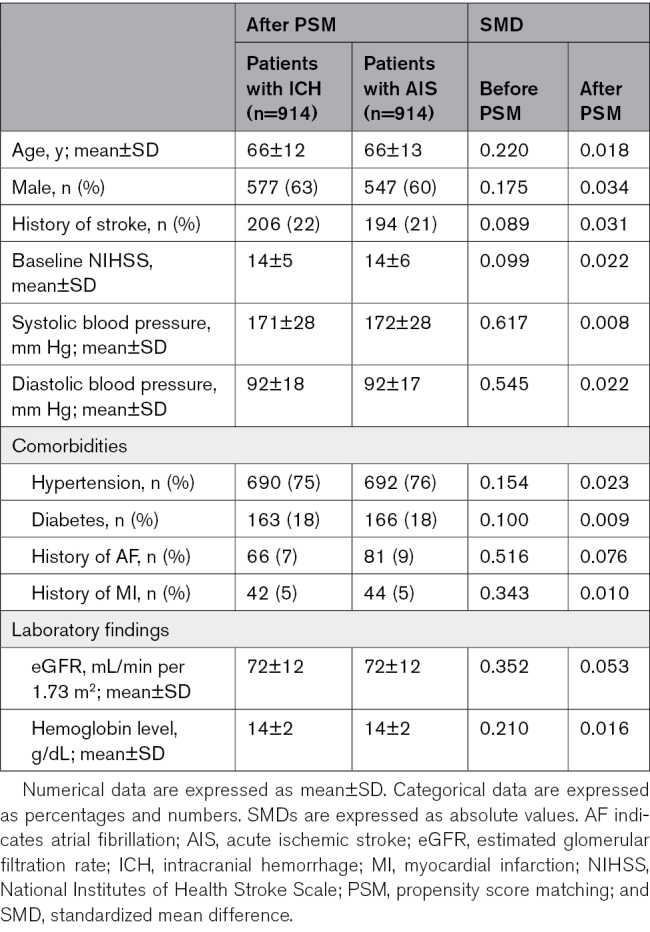
Patient Demographics

### Patients With SHS

Among 6548 adverse event terms, 248 terms were classified as cardiac events in the entire cohort. In the matched cohort, a total of 279 patients experienced 311 events classified as SHS or SHS-ICH. The median day of onset for both groups was identical, occurring on day 2 poststroke (interquartile range, 1–4 days). The predominant category was other arrhythmias/ECG abnormalities, encompassing 170 patients (55% of cases), followed by AF/AFL, HF/LV dysfunction, ACS/myocardial injury, and cardiorespiratory arrest.

Among those with ACS/myocardial injury, the majority (86%, 12 patients) were diagnosed with ACS. Similarly, for those presenting with HF/LV dysfunction, HF was the principal diagnosis in 90% (35 patients). Within the AF/AFL group, AF was the leading diagnosis (96%, 72 patients). Finally, in the category of other arrhythmia/ECG abnormalities, other arrhythmias were the most common (92%, 157 patients).

### Incidence of SHS

**Figure 2.**
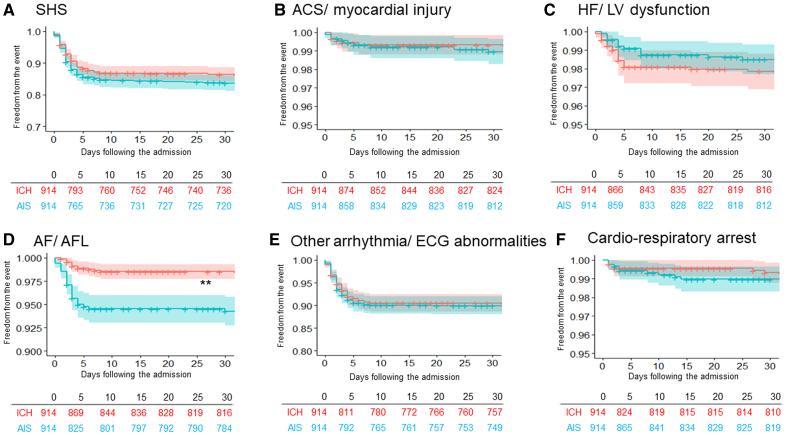
**Kaplan-Meier curves comparing the cumulative incidence of stroke-heart syndrome (SHS) and each manifestation. A**, SHS. **B**, Acute coronary syndrome (ACS)/myocardial injury. **C**, Heart failure (HF)/left ventricular (LV) dysfunction. **D**, Atrial fibrillation (AF)/atrial flutter (AFL). **E**, Other arrhythmia/ECG abnormalities. **F**, Cardiorespiratory arrest. Each plot is expressed as cumulative incidence freedom from the event with 95% CI. **Statistical significance (*P*<0.001). AIS indicates acute ischemic stroke; and ICH, intracranial hemorrhage.

Table 2 shows the incidence of SHS. Within the ICH group, 123 patients (13%) experienced SHS-ICH. Conversely, in the AIS group, SHS developed in 148 patients (16%). The Kaplan-Meier analysis results (Figure 2) show that the cumulative incidence of SHS tended to be lower in the ICH group compared with the AIS group although this difference did not reach statistical significance (Figure 2A; Table 3; the cumulative incidence freedom from the SHS event, 86.3% [95% CI, 84.1–88.6] versus 83.6% [95% CI, 81.2–86.0]; *P*=0.100).

**Table 2.**
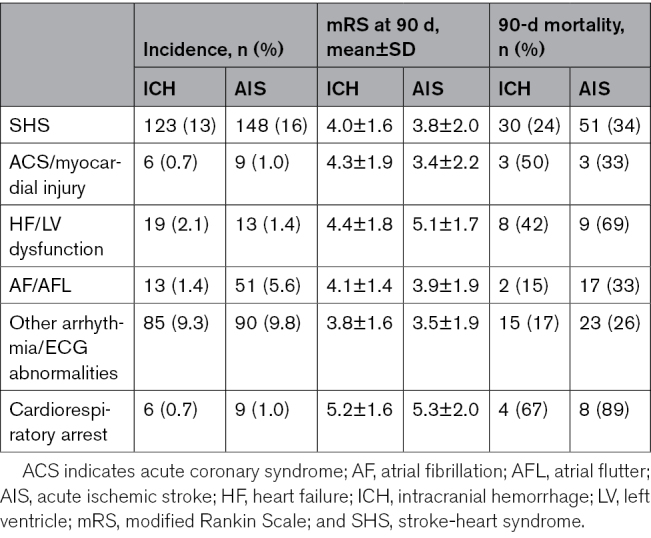
Incidence and Outcomes of SHS

**Table 3.**
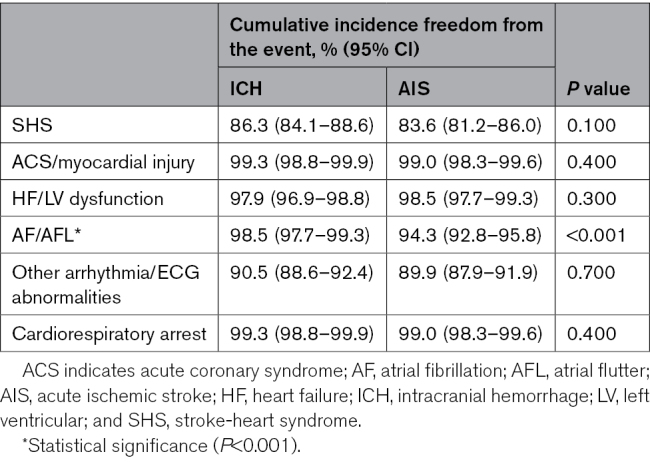
Cumulative Incidence Freedom From the Event in SHS

Across all manifestations, the cumulative incidence of ACS/myocardial injury, HF/LV dysfunction, other arrhythmia/ECG abnormalities, and cardiorespiratory arrest was comparable between the groups, as shown in Table 3. The cumulative incidence of AF/AFL was significantly lower in the ICH group than in the AIS group. The cumulative incidence freedom from AF/AFL was 98.5% (95% CI, 97.7–99.3) in the ICH group compared with 94.3% (95% CI, 92.8–95.8) in the AIS group (*P*<0.001; Figure 2D).

In the sensitivity analysis using the PSM model that included the history of HF, 513 patients were matched in each group. The results were consistent with those of the original matching cohort (Table S2). The incidence of AF/AFL remained significantly lower in the ICH group (*P*<0.001).

Table S3 compares the proportion of arrhythmia subcategories between groups. Regarding AF/AFL, most patients in both groups had de novo AF (ie, new incident AF). For other arrhythmias/ECG abnormalities, the proportions of bradyarrhythmias and ventricular arrhythmias were higher in the AIS group. Conversely, the proportion of ECG abnormalities was significantly higher in the ICH group (*P*=0.004).

### Prognostic Outcomes

In the ICH group, patients with SHS-ICH exhibited significantly higher modified Rankin Scale scores compared with those without SHS-ICH (Table 2; modified Rankin Scale, 4.0±1.6 for SHS-ICH versus 3.1±1.6 for non-SHS-ICH; *P*<0.001). A similar pattern was observed in the AIS group, where patients with SHS had worse neurological outcomes (modified Rankin Scale, 3.8±2.0 for SHS versus 2.8±1.8 for non-SHS; *P*<0.001).

The result in 90-day mortality also followed the trend. The mortality in patients with SHS-ICH was significantly higher than those without (Table 2; 24% versus 9%; *P*<0.001). Of note, cardiorespiratory arrest, ACS/myocardial injury, and HF/LV dysfunction exhibited higher mortality among all manifestations in ICH-SHS.

### Associations for 90-Day Mortality

Table 4 presents the associations for 90-day mortality in the ICH group after adjusting for multiple variables. SHS-ICH was independently associated with the outcome, with an adjusted odds ratio of 1.12 (95% CI, 1.06–1.19; *P*<0.001). In addition, age, male, and baseline National Institutes of Health Stroke Scale scores were also associated with poor outcomes. The sensitivity analysis that included a model with ICH volume as a covariate for the entire ICH cohort (n=1797) also demonstrated consistent results (adjusted odds ratio, 1.09 [95% CI, 1.01–1.19]; *P*=0.030).

**Table 4.**
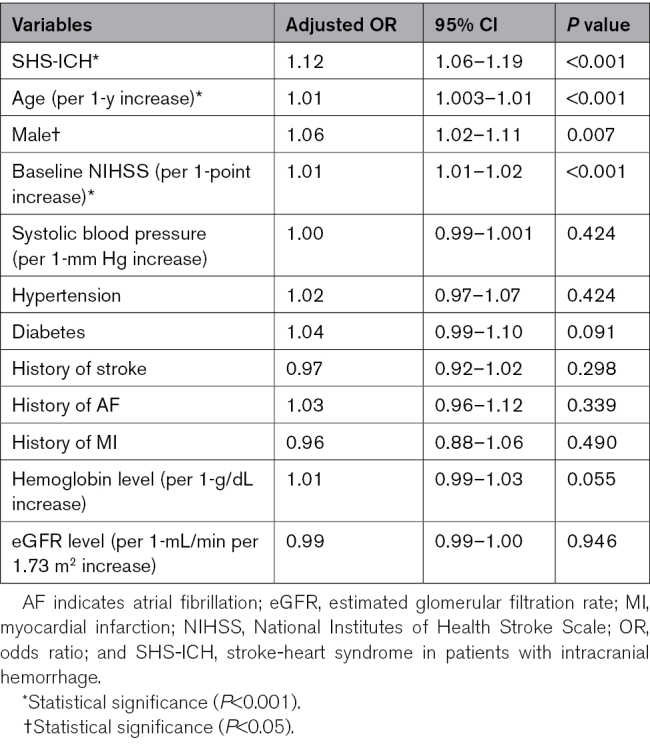
Multivariate Analysis Associated With 90-Day Mortality

## DISCUSSION

The key findings of this study are summarized as follows. First, while the total incidence of cardiac events was significantly lower in the ICH group compared with the AIS group, the incidences of events other than AF/AFL were comparable between the matched groups. Second, the incidence of AF/AFL was significantly lower in the ICH group than in the AIS group, even after adjusting for a history of AF in the PSM. Third, after adjusting for multiple variables, SHS-ICH was independently associated with 90-day mortality in patients with ICH.

### SHS in Patients With ICH

Given that the underlying mechanisms of SHS derive from cerebral injury, particularly involving the central autonomic network and insular cortex, neurogenic cardiac injury should manifest in patients with ICH as it does in those with AIS.^7^ Experimental research indicates that both AIS and ICH commonly precipitated systemic immune activation, characterized by elevated concentrations of proinflammatory cytokines and increased in oxidative stress markers, culminating in neurogenic cardiac dysfunction.^16,17^ Clinical studies have also shown that patients with ICH were prone to exhibit autonomic dysfunction, characterized by a reduction in heart rate variability and diminished baroreceptor sensitivity, similar to patients with AIS.^18,19^ Other studies have reported that in cases where the lesion involved the insular cortex, ECG abnormalities were frequently observed in patients with both ICH and AIS.^20,21^ One study also indicated that the incidence of myocardial injury, as characterized by elevated cardiac troponin levels, was comparable between patients with ICH and AIS, adversely impacting the prognosis in both conditions.^22^

While previous studies have indicated that cardiac events can occur in patients with ICH, there has been limited research on the incidence and specific types of cardiac events in these patients. Previous literature has reported the incidence of acute MI and HF during the acute stroke phase to be 0.3% to 4.3%^4,23,24^ and 3.8%,^24^ respectively. Nonetheless, analyses encompassing the comprehensive incidence of cardiac events in patients with ICH are scarce. Our study contributes new information on this issue, showing that the incidence of SHS-ICH at 13% was much higher than the 4.1% reported in earlier research.^24^ This discrepancy likely stems from differences in the study populations. While the prior study predominantly focused on severe manifestations such as acute MI, HF, and lethal arrhythmias,^24^ our analysis includes AF/AFL and less severe manifestations, such as LV dysfunction and ECG abnormalities. Despite these insights, the overall scarcity of reports in this field highlights the need for future studies to understand the SHS entity.

### SHS in ICH Versus Ischemic Stroke

In our study, we uniquely compared the incidence of cardiac events between patients with ICH and matched patients with AIS. The incidence disparity was exclusively noted in AF/AFL, suggesting the difference in underlying conditions (ICH versus AIS) rather than the difference in the mechanism for developing cardiac events. Given that AF is a major contributor to AIS but not to ICH, AF comorbidity is much more common in AIS than in ICH. Furthermore, previous research suggested that the incidence of AF poststroke was more prevalent in AIS than in ICH.^25,26^ Even from the perspective of clinical practice, clinicians also tend to actively investigate evidence of AF in patients with AIS compared with patients with ICH.^27^ Cardiac dysfunction detected after ischemic stroke is common.^28^ However, it is often challenging to determine whether this dysfunction existed before the stroke and was only diagnosed during hospitalization and subsequent investigations. In many ischemic stroke cases, the cardiac pathology may be the cause of the stroke, rather than a consequence. In contrast, cardiac causes of hemorrhagic stroke are relatively rare, except in cases of infective endocarditis or the use of anticoagulant medications for known AF.

The differences in the incidence of SHS between ICH and AIS may also be partly due to variations in initial and posthospital medical care. Nearly all patients with AIS undergo echocardiography and cardiac monitoring as part of their stroke work-up. In contrast, not all patients with ICH receive an echocardiogram, and most do not undergo cardiac monitoring. We have utilized PSM to minimize the selection and surveillance biases. However, due to the retrospective nature of the data, the biases may have underestimated the rates of SHS observed in ICH compared with AIS. We have also performed sensitivity analysis to minimize the biases in our results.

In addition, we observed that the proportion of ECG abnormalities was significantly higher in patients with ICH although the overall incidence of other arrhythmias/ECG abnormalities was comparable between groups. While ECG changes mimicking myocardial ischemia tend to be more evident in subarachnoid hemorrhage compared with other subtypes of stroke, the findings for ICH and AIS still remain uncertain.^29^ It is challenging to conclude whether these differences were due to the etiological differences between the 2 conditions due to our limited sample size.

### Clinical Implications

Our study sheds light on the impact of cardiac events associated with ICH (ICH-SHS) on poor short-term prognosis, mirroring the effects observed in patients with AIS. Notably, the incidences of manifestations other than AF/AFL were similar between the matched ICH and AIS cohorts. This similarity highlights the critical need for prompt identification and management of cardiac events in patients with ICH, just as in patients with AIS. Given that patients with ICH tend to develop more pronounced disturbances in consciousness levels compared with those with AIS,^30^ detecting new cardiac complications can be more challenging. Even in such situations, our data underscore the importance of remaining vigilant for these complications.

### Limitations

Our study has several limitations. First, the identification of cardiac events was dependent on the documentation of adverse events reporting across various clinical trials included in the VISTA database, rather than direct examination of medical records. This method may lead to underestimation of the true frequency of SHS. Of note, the standard of practice for ICH does not involve active cardiac screening for AF and other cardiac abnormalities unlike in AIS. Thus, our data may contain reporting and surveillance biases. In addition, our definition of myocardial injury cannot distinguish between acute and chronic myocardial injury as recommended by contemporary guidelines. This is due to limited data on the dynamic changes through serial measurements of high-sensitivity troponin assays for the diagnosis.^31^ Of note, our data were from clinical stroke trials completed before the contemporary ACS guidelines. Furthermore, the data in our study were collected when high-sensitivity troponin assays were not fully implemented. Hence, the incidence of myocardial injury might differ from the current clinical settings. Nevertheless, in our data, the number of cases categorized as myocardial injury is small, and we think that its impact would be minimal. Second, the retrospective design of our analysis was limited by the availability of certain variables within the database. For example, our study did not include information on racial/ethnic differences and the anatomic location of stroke lesions, both of which could have influenced the incidence of SHS.^32^ Third, the AIS cohort in our study was derived through matching with the ICH cohort using PSM. This approach may not fully capture the typical AIS population, potentially skewing toward a high-risk profile due to the matching criteria.

### Conclusions

Cardiac events are common and negatively affect prognosis in patients with ICH, just as seen in AIS.

## ARTICLE INFORMATION

### Acknowledgments

The authors thank all Virtual International Stroke Trials Archive collaborators, who contributed previous clinical trial data.

### Sources of Funding

This study was funded by the Dowager Countess Eleanor Peel Trust, United Kingdom.

### Disclosures

Dr Dawson received speaker fees from AstraZeneca, Bayer, Boehringer Ingelheim, Bristol Myers Squibb, Daiichi Sankyo, Medtronic, and Pfizer and a travel fee from MicroTransponder, Inc. Dr Dawson also received investigator-initiated research funding grant from the Stroke Association United Kingdom. Dr Lip is a consultant and a speaker for BMS/Pfizer, Boehringer Ingelheim, Daiichi Sankyo, and Anthos. No fees are received personally. He is a National Institute for Health and Care Research Senior Investigator and a co-principal investigator of the AFFIRMO project on multimorbidity in AF (grant agreement 899871), the TARGET project on digital twins for personalized management of atrial fibrillation and stroke (grant agreement 101136244), and the ARISTOTELES project on artificial intelligence for management of chronic long-term conditions (grant agreement 101080189), which are all funded by the Horizon Europe Research and Innovation Programme of the European Union. The other authors report no conflicts.

### Supplemental Material

Tables S1–S3

Figure S1

## APPENDIX

VISTA-Acute Steering Committee Members: K.R. Lees (Chair), A. Alexandrov, P.M. Bath, E. Bluhmki, N. Bornstein, C. Chen, L. Claesson, J. Curram, S.M. Davis, H-C. Diener, G. Donnan, M. Fisher, M. Ginsberg, B. Gregson, J. Grotta, W. Hacke, M.G. Hennerici, M. Hommel, M. Kaste, P. Lyden, J. Marler, K. Muir, C. Roffe, R. Sacco, A. Shuaib, P. Teal, N. Venketasubramanian, N.G. Wahlgren, and S. Warach.

## Supplementary Material
