# Supplementary material for: Incidence and Outcomes of Patients With Early Cardiac Complications After Intracerebral Hemorrhage: A Report From VISTA
Source: Stroke. 2024 Oct 2;55(11):2669–76. doi: 10.1161/STROKEAHA.124.048189 (PMC11518970; doi:10.1161/STROKEAHA.124.048189)
Supplement: Supplementary file 1 [file str-55-2669-s001.pdf]

SUPPLEMENTARY MATERIALS

Figure S1. Comparison of Standardised Mean Difference

Table S1. Patient Demographics in the Whole Cohort

Table S2. Cumulative Incidence Freedom from the Event in Stroke-Heart Syndrome in the Model Including the History of HF

Table S3. The Comparison of Arrhythmia Subcategories

Figure S1

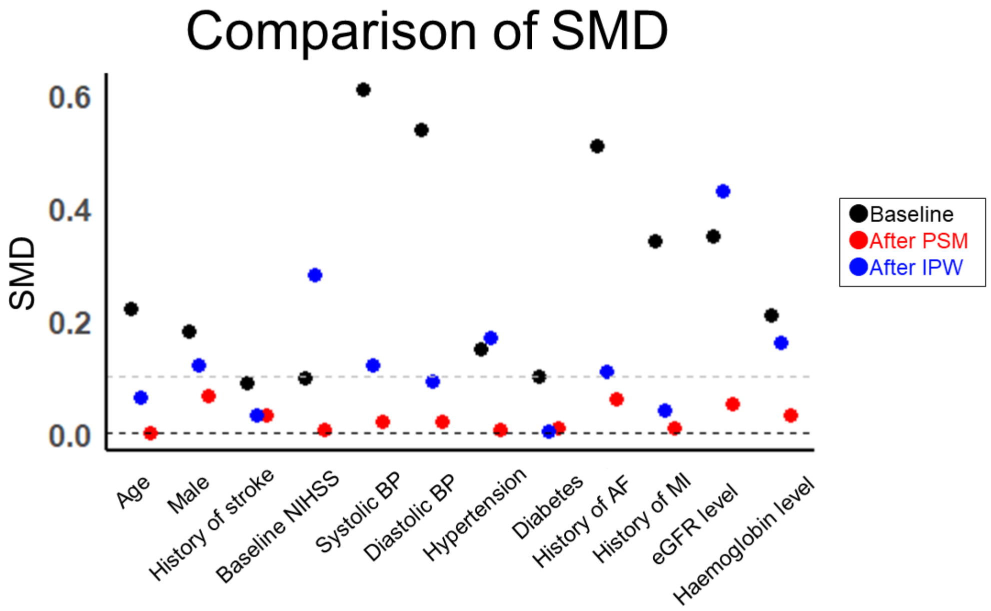

SMDs were expressed as the absolute values.
AF, atrial fibrillation; BP, blood pressure; eGFR, estimated glomerular filtration rate; IPW, inverse probability weighting; MI, myocardial infarction; NIHSS, National Institutes of Health Stroke Scale; PSM, propensity score matching; SMD, standardised mean difference

Table S1. Patient Demographics in the Whole Cohort

	Patients with ICH (n=1,797)	Patients with AIS (n=15,054)	SMD
Age (years), mean±SD [0]	66±12	69±12	0.140
Male, n (%) [0]	1,141 (63)	8,353 (55)	0.136
History of stroke, n (%) [0.46]	338 (19)	3,305 (22)	0.068
Baseline NIHSS, mean±SD [26]	14±6	13±6	0.170
Systolic blood pressure (mmHg), mean±SD [0.54]	171±26	157±25	0.529
Diastolic blood pressure (mmHg), mean±SD [0.55]	92±17	85±15	0.456
ICH volume (ml), median (Q1, Q3) [65]	17 (8, 32)	-	-
**Comorbidities**			
Hypertension, n (%) [5]	1,238 (71)	9,701 (68)	0.073
Diabetes, n (%) [0.05]	295 (16)	3,237 (22)	0.130
History of AF, n (%) [13]	124 (7)	3,164 (24)	0.483
History of MI, n (%) [29]	50 (5)	1,477 (13)	0.310
History of HF, n (%) [54]	18 (3)	755 (11)	0.305
**Laboratory findings**			
eGFR (ml/min/1.73m^2), mean±SD [32]	72±12	68±12	0.341
Haemoglobin level (g/dl), mean±SD [39]	14±2	14±2	0.164

Numerical data are expressed as mean±SD or median (interquartile range; first quartile, third quartile). Categorical data are expressed as percentages and numbers. SMDs are expressed as the absolute values. [] indicates missing rate (%).

AF, atrial fibrillation; AIS, acute ischaemic stroke; eGFR, estimated glomerular filtration rate; HF, heart failure; ICH, intracranial haemorrhage; MI, myocardial infarction; NIHSS, National Institutes of Health Stroke Scale; PSM, propensity score matching; SD, standard deviation; SMD, standardised mean difference

Table S2. Cumulative Incidence Freedom from the Event in Stroke-Heart Syndrome in the Model Including the History of HF

	Cumulative incidence freedom from the event, % (95%CI)		P-value
	ICH (n=513)	AIS (n=513)	
SHS	85.3 (82.3-88.5)	84.3 (81.2-87.5)	0.600
ACS/myocardial injury	99.0 (98.2-99.9)	99.4 (98.7-100)	0.500
HF/LV dysfunction	96.9 (95.4-98.5)	98.2 (97.1-99.4)	0.200
AF/AFL **	99.2 (98.4-100)	94.6 (92.7-96.6)	<0.001
Other arrhythmia/ECG abnormalities	90.0 (87.4-92.6)	90.0 (87.4-92.6)	>0.999
Cardio-respiratory arrest	99.2 (98.4-100)	99.6 (98.0-100)	0.400

ACS, acute coronary syndrome; AF, atrial fibrillation, AFL, atrial flutter; AIS, acute ischaemic stroke; ECG, electrocardiogram; HF, heart failure; ICH, intracranial haemorrhage; LV, left ventricular; SHS, stroke-heart syndrome. Asterisk (**) indicates statistical significance (P<0.001).

Table S3. The Comparison of Arrhythmia Subcategories

	Patients with ICH	Patients with AIS	P-value
AF/AFL	**(n=13)**	**(n=51)**	
AF/AFL without the history of AF	10 (77)	46 (90)	0.411
Other arrhythmia/ECG abnormalities	**(n=85)**	**(n=90)**	
Bradyarrhythmias, n (%)	30 (35)	41 (46)	0.219
Supraventricular arrhythmias other than AF/ AFL, n (%)	15 (18)	12 (13)	0.561
Ventricular arrhythmias, n (%)	25 (29)	35 (39)	0.245
Other arrhythmias, n (%)	5 (6)	2 (2)	0.395
ECG abnormalities, n (%) *	10 (12)	0	0.004

AF, atrial fibrillation, AFL, atrial flutter; AIS, acute ischaemic stroke; ECG, electrocardiogram; ICH, intracranial haemorrhage;. Asterisk (*) indicates statistical significance (P<0.05).
